# Identification of Peritrophins and Antiviral Effect of *Bm01504* against BmNPV in the Silkworm, *Bombyx mori*

**DOI:** 10.3390/ijms21217973

**Published:** 2020-10-27

**Authors:** Xu-Le Zha, Xin-Bo Yu, Hong-Yan Zhang, Han Wang, Xian-Zhi Huang, Yi-Hong Shen, Cheng Lu

**Affiliations:** 1 State Key Laboratory of Silkworm Genome Biology, Southwest University, Beibei, Chongqing 400715, China; zhaxl1005@126.com (X.-L.Z.); yuxinbo1234@163.com (X.-B.Y.); hongyanzhang0321@163.com (H.-Y.Z.); wh18223@163.com (H.W.); 2 Science and Technology Department, Southwest University, Chongqing 400715, China; hxz1166@swu.edu.cn

**Keywords:** *Bombyx mori*, peritrophic membrane, peritrophin, *Bombyx mori nucleopolyhedrovirus*, gene editing

## Abstract

The insect midgut secretes a semi-permeable, acellular peritrophic membrane (PM) that maintains intestinal structure, promotes digestion, and protects the midgut from food particles and pathogenic microorganisms. Peritrophin is an important PM protein (PMP) in the PM. Here, we identified 11 peritrophins with 1–16 chitin binding domains (CBDs) comprising 50–56 amino acid residues. Multiple CBDs in the same peritrophin clustered together, rather than by species. The CBD contained six highly conserved cysteine residues, with the key feature of amino acids between them being CX_11-15_CX_5_CX_9-14_CX_11-12_CX_6-7_C. Peritrophins with 2 and 4 CBDs (*Bm09641* and *Bm01504*, respectively), and with 1, 8, and 16 CBDs (*Bm11851*, *Bm00185*, and *Bm01491*, respectively) were mainly expressed in the anterior midgut, and throughout the midgut, respectively. Survival rates of transgenic silkworms with Bm01504 overexpression (Bm01504-OE) and knockout (Bm01504-KO) infected with *B. mori* *nucleopolyhedrovirus* (BmNPV) were significantly higher and lower, whereas expression of the key viral gene, *p10*, were lower and higher, respectively, compared with wild type (WT). Therefore, Bm01504-OE and Bm01504-KO transgenic silkworms were more and less resistant, respectively, to BmNPV. *Bm01504* plays important roles in resisting BmNPV invasion. We provide a new perspective for studying PM function, and reveal how the silkworm midgut resists invasive exogenous pathogenic microorganisms.

## 1. Introduction

Insect midgut secretes a semi-permeable peritrophic membrane (PM) that protects midgut epithelial cells from damage by food particles, toxic compounds and invasive pathogenic microorganisms [1,2,3,4,5,6]. Recently, biological or chemical methods have been successfully used to destroy the PM structure as a means of pest control [7,8,9,10,11,12]. The PM consists of chitin fibrils associated with glycoproteins and proteoglycans [13,14]. Shi et al. (2004) proposed a structural model of PM, in which chitin microfibrils consisting of 20–400 chitin chains assemble into chitin bundles to form the backbone of the PM network, and PM protein (PMP) adheres to chitin via non-covalent bonding or chitin binding domains (CBD) [15]. According to the degree of binding with PM, PMPs can be categorized into four classes. PMPs of class I–III can be removed from the PM by physiological buffers, mild detergents, such as Triton X-100, and strong detergents, such as urea, respectively; however, class IV proteins cannot be removed by these three methods. Among them, class III is also named peritrophins that contains at least one CBD [16].

Two major chitin-binding proteins belonging to pfam00379 and pfam01607 families have been identified in insects based on distinct sequence characteristics. The chitin-binding proteins of pfam00379 family contains the histidine-rich, cysteine-deficient CBD with the extended R&R consensus (Chitin_Bind_4), a 68-amino-acid motif that is exclusively found in cuticular proteins. The pfam01607 (Carbohydrate Binding Module family14; CBM_14) family contains a type 2 CBD (ChtBD2) that is found in peritrophins, cuticle proteins analogous to peritrophins (CPAPs), chitinases (CHTs), and chitin deacetylases (CDAs) [17,18]. Among them, CBD in peritrophins comprise peritrophin-A, -B, and -C, with structures characterized by 6, 8, and 10 cysteine residues, and with 48–57, 81–88 and 121–122 amino acids, respectively, in which the respective amino acid features of cysteine residues are CX_13-18_CX_5_CX_9-11_CX_10-13_CX_7-8_C, CX_12-14_CX_18-21_CX_10-18_CX_12_CX_2_CX_8_C_7-12_C and CX_17_CX_9-10_CX_14_CX_9_CX_8-9_CX_19_C_9-11_CX_14_CX_11_C, respectively. Although peritrophin-A is found in various insects, peritrophin-B, -C are only detected in dipteran insects [16,19]. Previous studies have identified 1–14 CBDs in *Tribolium castaneum* and *Manduca sexta* peritrophins, and 1–24 CBDs in *Bombyx mori* peritrophins [17,18,20]. Based on the composition of CBD and mucin domain (MD), Toprak et al. (2010) classified peritrophins into four types and predicted the function of each. Simple peritrophins (1–2 CBDs) may be involved in protecting the midgut from exogenous microorganisms. The binary peritrophins (2 CBDs + 1 MD) and the complex peritrophins (≥3 CBDs + ≥1 MD) may play important roles in maintaining the PM structure and inhibiting proteolysis. Repetitive peritrophins (≥3 CBDs) may affect the morphology, tensile strength, elasticity, and permeability of the PM [16].

Over 30 peritrophins have been identified using cDNA libraries or genome databases [15,17,18,20,21,22,23,24,25,26,27,28,29,30,31,32,33,34,35]. In addition, several peritrophin-like genes that are similar to insect peritrophins have been identified in crustaceans [36,37,38,39,40]. The functions of peritrophins have recently attracted considerable attention given their potentially important biological roles with respect to the survival of insects. Inhibition of two PMP coding genes, *TcPMP3* and *TcPMP5-B*, was found to destroy the structure of PM, resulting in fat body depletion, growth arrest, molting defects, and mortality of *Tribolium castaneum* [41]. Moreover, RNAi for *SfPER* significantly decreased pupal weight and adult emergence rates of *Spodoptera frugiperda* [8]. Knockdown of *PpPer1* in *Phlebotomus papatasi* resulted in a 39% increase in parasite load at 48 h [42]. Similar results were observed in crustaceans, with inhibition of the peritrophin-like *EsPT* gene in *Eriocheir sinensis* increasing the number of *Vibrio parahaemolyticus* [37]. These results suggest that peritrophins are a component of the PM and are likely involved in the function of the PM in host digestion, nutrient absorption or as barrier against invasion by exogenous pathogenic microorganisms.

The role of peritrophins in the resistance to invasion by exogenous pathogenic microorganisms has not been studied in detail in silkworm. The completion of a silkworm genome database has facilitated our investigation on the function of peritrophins. A homology search performed by Li et al. (2019) identified 46 proteins with the ChtBD2 domain in the silkworm genome database, which included 26 CPAPs, 13 peritrophins, 4 CHTs, and 3 CDAs [20]. Here, we identified 11 peritrophins in the silkworm genome database by a bioinformatics method. The *Bm01504* gene was overexpressed and knocked out to study its role in resisting *B. mori nucleopolyhedrovirus* (BmNPV) invasion. This study provides a new perspective for studying the function of the PM, as well as for analyzing resistance of the silkworm midgut to the invasion of exogenous pathogenic microorganisms.

## 2. Results

### 2.1. Identification and Bioinformatics Analysis of Peritrophins in Bombyx mori

A total of 40 proteins containing the peritrophin-A domain were identified in the silkworm genome database based on the Hidden Markov Model (HMM) of the CBM_14 family (Table S1). According to the type of conserved domain and evolutionary characteristics of the CBD, these genes were divided into CHTs (*n* = 3), mucins (MUC; *n* = 4), CDAs (*n* = 3), CPAP1 (*n* = 13), CPAP3 (*n* = 6), and peritrophins (*n* = 11) (Table 1). The numbers of CBDs in peritrophins varied from 1 to 16, suggesting that these proteins have different evolutionary characteristics and functions.

**Figure 1:**
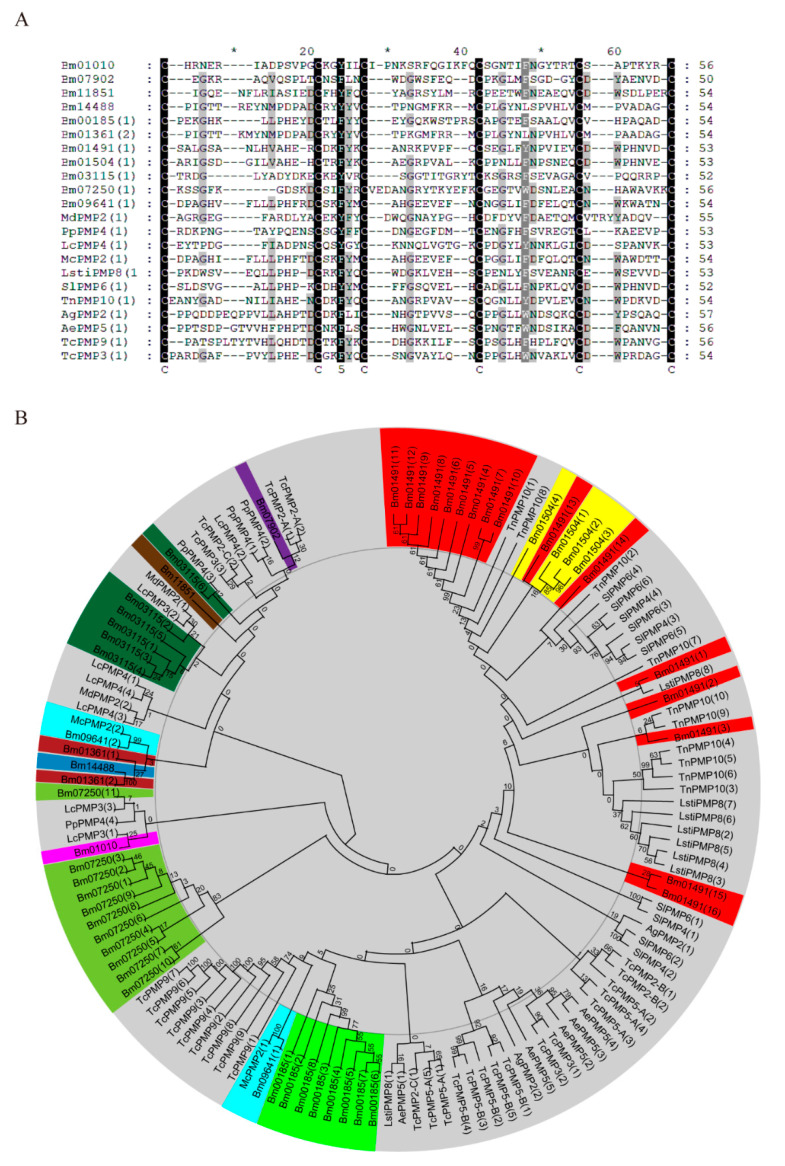
Phylogenetic analysis and multiple sequence alignment of CBDs in peritrophins. Species included *Aedes aegypti* (Aa), *Anopheles gambiae* (Ag), *Bombyx mori* (Bm), *Loxostege sticticalis Linne* (Lsti), *Lucilia cuprina* (Lc), *Mamestra configurata* (Mc), *Mayetiola destructor* (Md), *Phlebotomus papatasi* (Pp), *Spodoptera litura* (Sl), *Tribolium castaneum* (Tc), *Trichoplusia ni* (Tn). (**A**) Amino acid sequence alignment of first CBD in peritrophins. The CBD sequence comprised 52–56 amino acid residues, and a consensus of conserved cysteines (C) and spaces (X): CX_11-15_CX_5_CX_9-14_CX_11-12_ CX_6-7_C. Black shading indicates cysteine and other conserved residues in CBDs. The less-conserved amino acids are shown in shades of gray; (**B**) Phylogenetic tree of CBDs from several species. *Bombyx mori* peritrophins include Bm01491 (red), Bm11851 (brown), Bm09641 (sky blue), Bm01504 (yellow), Bm00185 (lime), Bm07250 (yellow green), Bm01010 (fuchsia), Bm07902 (purple), Bm14488 (cyan), Bm01361 (dark red), Bm03115 (dark green). The accession numbers of all proteins are listed in Table S3.

Multiple sequence alignment of the first CBD in peritrophins from different insects showed that the CBD in peritrophins comprised 50–56 amino acid residues, six highly conserved cysteine residues, and a consistent number of amino acids among the cysteine residues, namely, CX_11-15_CX_5_CX_9-14_CX_11-12_CX_6-7_C (Figure 1A). Phylogenetic analysis did not reveal any clear clustering by species, with many CBDs from the same protein tending to cluster together. We speculated that although the CBD appeared before the diversification of insects, the events that led to an increase in the number of CBDs in peritrophins occurred after insect species diversification (Figure 1B). In contrast to other peritrophins, the CBDs of Bm09641 clustered with that of McPMP2 in *Mamestra configurata*. This mode of evolution is similar to that of the CBD in CPAP3, suggesting that the number of CBDs in peritrophins were amplified before species differentiation (Figure 1B). Phylogenetic analysis also revealed that the CBDs of Bm01504 and Bm01491 had 48% similarity (Table S2), and both genes were located on chromosome 21 (Table 1). Moreover, the CBDs of Bm01361 and Bm14488 showed 90% similarity (Table S2). Therefore, *Bm01504* and *Bm14488* may be replicated from *Bm01491* and *Bm01361*, respectively. Altogether, these results demonstrated that peritrophins are evolutionarily diverse.

### 2.2. Expression Patterns of Peritrophins in Silkworm, Bombyx mori

**Figure 2:**
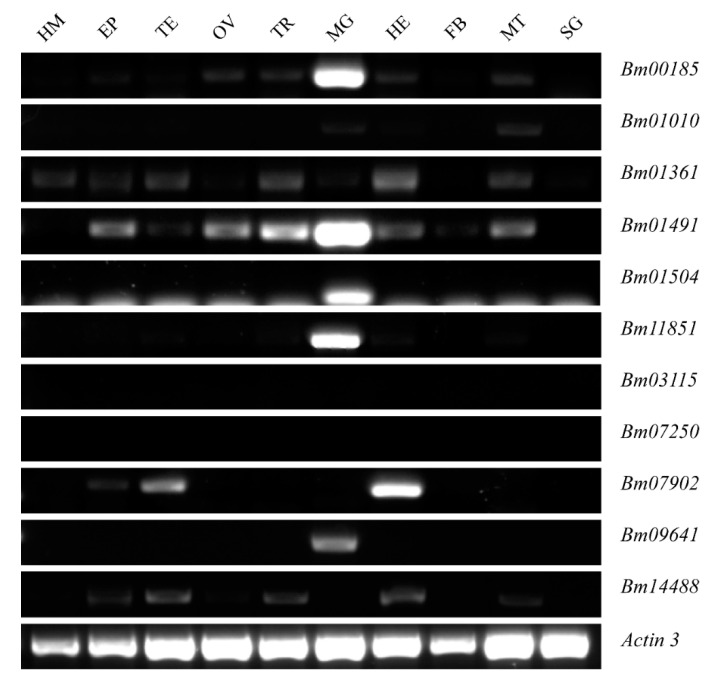
The expression levels of the 11 identified peritrophins in various tissues at 3rd day of the fifth instar larvae. *Bm03115*, *Bm07250*, *Bm07902*, and *Bm14488* were not detected in the midgut. *Bm01010*, *Bm01361* showed very low expression in the midgut. *Bm11851*, *Bm09641*, *Bm01504*, *Bm00185*, and *Bm01491* were highly or specifically expressed in the midgut. Actin 3 served as an internal control. EP, Epidermis; FB, Fat body; HE, Head; HM, Hemolymph; MG, Midgut; MT, Malpighian tubule; OV, Ovary, SG, Silk gland; TE, Testis; TR, Trachea.

The expression of 11 peritrophins in different tissues on the 3rd day of the fifth instar larvae of *Bombyx mori* were analyzed at the messenger RNA (mRNA) levels by using semi-quantitative reverse transcription-polymerase chain reaction (RT-PCR). *Bm11851*, *Bm09641*, *Bm01504*, *Bm00185*, and *Bm01491* were found to be highly expressed in the midgut, *Bm01010*, *Bm01361* were expressed at low levels in the midgut, whereas the expression of the other four genes in the midgut was barely detectable (Figure 2).

**Figure 3:**
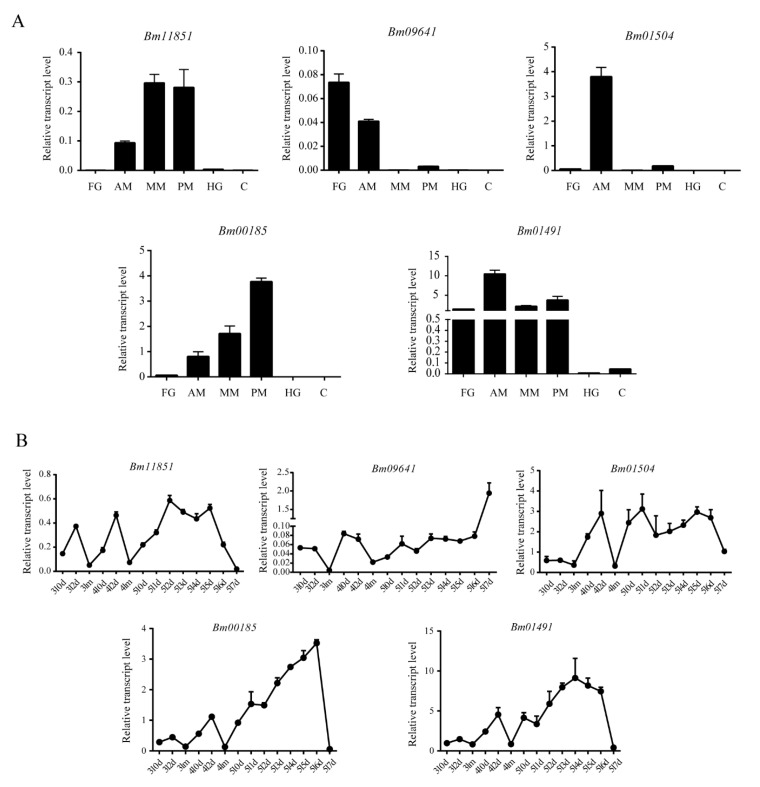
Temporal and spatial expression patterns of peritrophins in *Bombyx mori*. (**A**) Expression of peritrophins in different regions of the midgut. AM, Anterior midgut; FG, Foregut; HG, Hindgut; MM, Middle midgut; PM, Posterior midgut; (**B**) Expression of peritrophins in the midgut from the third instar to 7th day of the fifth instar. 3l, Third instar of larval stage; 4l, Fourth instar of larval stage; 5l, Fifth instar of larval stage; m, Molt; nd, Day n.

Quantitative RT-PCR (qRT-PCR) further showed that *Bm09641* and *Bm01504* (comprising 2 and 4 CBDs, respectively,) were expressed in the anterior midgut, whereas *Bm00185*, *Bm01491*, and *Bm11851* (with 8, 16, and 1 CBDs, respectively) were expressed throughout the midgut (Figure 3A). Overall, peritrophins with different amounts of CBDs were found to have diverse expression patterns across the midgut, suggesting that they may have different functions. The developmental expression profiles of the peritrophins from the third instar to 7th day of the fifth instar showed that peritrophins were more abundant during the feeding than the molting stage. The expression levels of peritrophins gradually increased with the development (Figure 3B), indicating that peritrophins play an important role in food intake and digestion. The expression of *Bm11851*, *Bm01504*, *Bm00185*, and *Bm01491* decreased at 7th day of the fifth instar, which could be related to dissociation of the larval midgut during metamorphosis (Figure 3B). Moreover, the expression of *Bm09641* notably increased on 7th day of the fifth instar (Figure 3B), indicating that this particular peritrophin may also play an important role in metamorphosis.

### 2.3. Generation of Bm01504 Overexpression (Bm01504-OE) in Transgenic Silkworms

**Figure 4:**
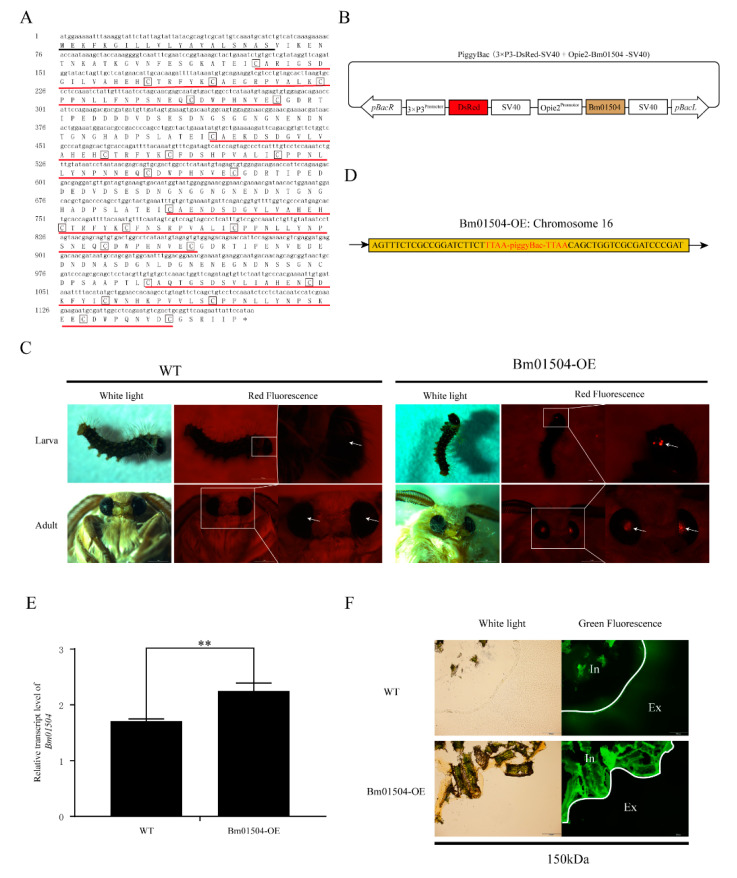
Generation of Bm01504 overexpression (Bm01504-OE) transgenic silkworms. (**A**) The nucleotide sequence of cDNA for *Bm01504* and its deduced amino acid sequence. The black line indicates the signal peptide sequence, the red line indicates the four CBDs, and conserved cysteine residues are boxed; (**B**) Schematic diagram of the piggyBac (3×P3-Discosoma sp. red fluorescent protein (DsRed)-SV40+ Opie2-Bm01504-SV40) transgenic vector. *Bm01504* was driven by Opie2 promoter, DsRed was driven by the 3×P3 promoter and SV40 was used for transcription termination signals. Hyperactive promoter containing three binding sites for Pax-6 homodimers in front of TATA box are indicated by 3×P3 promoter. DsRed, *Discosoma* sp. Red Fluorescent Protein. Opie2 promoter, *Orgyia pseudotsugata multicapsid nucleopolyhedrovirus* (OpMNPV) immediate-early 2 (ie2) promoter. SV40, terminator of *Simian virus 40*. pBacL and pBacR indicate the left and right terminal inverted repeats, respectively; (**C**) Images of larvae and adults of transgenic silkworm with white light and red fluorescence. WT, Wild type. White arrows indicate positions of eyes in larvae and adults; (**D**) Insertion sites in Bm01504-OE transgenic silkworms analyzed using inverse PCR; (**E**) The messenger RNA (mRNA) level of *Bm01504* in Bm01504-OE transgenic silkworms was determined by qRT-PCR. Values with *p* < 0.05 were considered statistically significant (** *p* < 0.01); (**F**) The anterior peritrophic membrane (PM) permeability of Bm01504-OE transgenic silkworms was evaluated using Fluoresceine isothiocyanate (FITC)-dextran (150 kDa) using fluorescence microscopy (100×). In, Internal. Ex, External. White lines show the localization of the PM.

The microarray data of Cheng et al. (2016) showed that the expression levels of *Bm01504*, *Bm09641* and *Bm11851* were significantly up-regulated when the silkworms were infected with BmNPV, whereas no significant change was observed in the expression of *Bm00185* and *Bm01491* [43]. Since, among these peritrophins, *Bm01504* showed the most significant change upon infection, this gene was selected to further investigate the role of peritrophins in BmNPV invasion of the midgut. We cloned full-length of *Bm01504* (Figure 4A). Sequencing results showed that the open reading frame of *Bm01504* was 1179 bp, encoding 392 amino acids. 20-amino acid signal peptide in the deduced protein was predicted by SignalP 5.0 (http://www.cbs.dtu.dk/services/SignalP/). Four conserved CBDs were predicted using pFam (http://pfam.xfam.org/). The piggyBac (3×P3-Discosoma sp. red fluorescent protein (DsRed)-SV40+Opie2-Bm01504-SV40) transgenic vector was constructed (Figure 4B). The transgenic vector and the helper vector were co-injected into 305 embryos to generate G0 offspring, which were then backcrossed with the G1 progeny. Transgenic silkworms of Bm01504 overexpression (Bm01504-OE) with red eyes from the G1 generation were screened by fluorescence microscopy (Figure 4C), and the insertion site was analyzed using inverse PCR. The results indicated that the gene was inserted into the silkworm genome at sole insertion site located in an intergenic region on chromosome 16 (Figure 4D). The expression level of *Bm01504* was detected in Bm01504-OE transgenic silkworms and WT silkworms. The results of qRT-PCR indicated that *Bm01504* had been successfully overexpressed in transgenic lines (Figure 4E). The permeability of the PM of the transgenic silkworms was evaluated using Fluoresceine isothiocyanate (FITC)-dextran, which revealed that 150 kDa dextran could pass through the anterior PM of WT, whereas it could not pass the anterior PM of Bm01504-OE (Figure 4F). These results indicate that Bm01504-OE transgenic silkworms reduced PM permeability.

### 2.4. Generation of Bm01504 Knockout (Bm01504-KO) in Transgenic Silkworms

**Figure 5:**
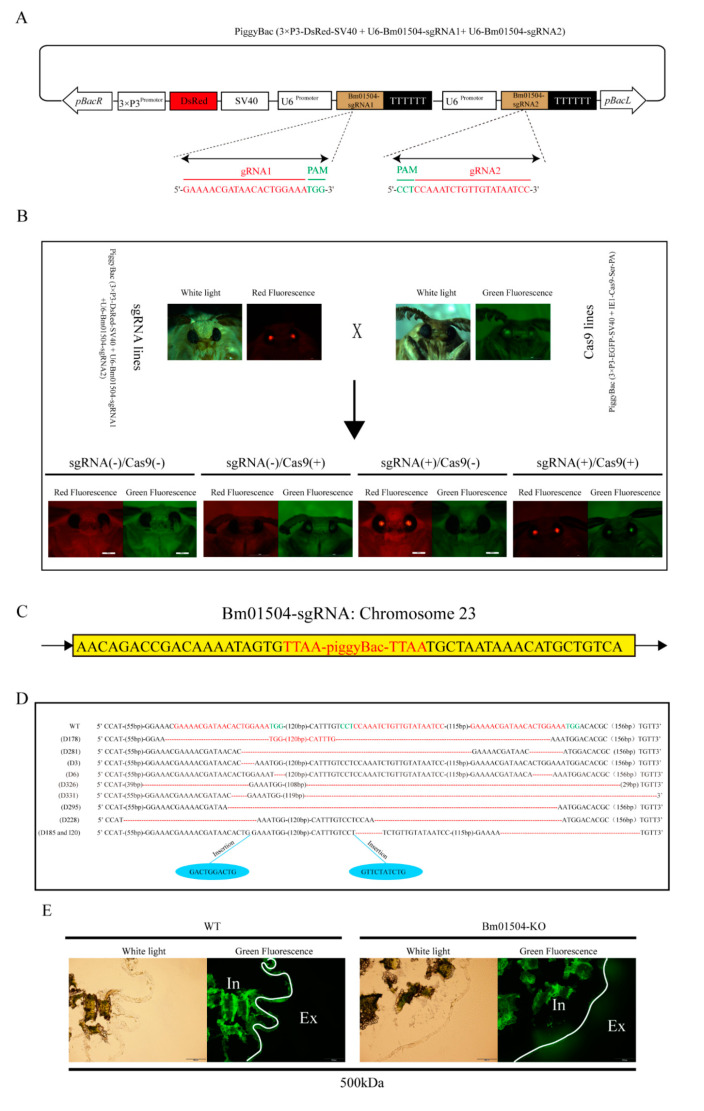
Generation of Bm01504 knockout (Bm01504-KO) transgenic silkworms. (**A**) Schematic diagram of the piggyBac (3×P3-DsRed-SV40 + U6-Bm01504-sgRNA1 + U6-Bm01504-sgRNA2) transgenic vector. DsRed was driven using 3×P3 promoter and SV40 was used for termination signals of transcription. 3×P3 indicate a hyperactive promoter containing three binding sites for Pax-6 homodimers in front of a TATA box. DsRed, Discosoma sp. Red Fluorescent Protein. SV40, Terminator of *Simian virus 40*. U6 promoter regulated the expression of Bm01504-sgRNA. U6, U6 snRNA promoter in *Bombyx mori*. Bm01504-sgRNA, gRNA targets of *Bm01504* gene designed by CRISPR direct. pBacL and pBacR, left and right terminal inverted repeats. Protospacer adjacent motif (PAM) sequence is in green; (**B**) Images of transgenic silkworm in white light, green and red fluorescence. G1 generation of Cas9 and sgRNA lines screened by fluorescence microscopy. The G2 generation of four transgenic hybrid lines, namely, Cas9 (–) / sgRNA (–), Cas9 (+) / sgRNA (–), Cas9 (+) /sgRNA (–), and Cas9 (+) / sgRNA (+) generated by G1 hybridization. Cas9 (+) / sgRNA (+) is named Bm01504-KO; (**C**) Insertion sites in Bm01504-sgRNA transgenic silkworms analyzed using inverse PCR; (**D**) Knockout effect of *Bm01504* in Bm01504-KO transgenic silkworms. Sequence of *Bm01504* target site is labeled in red. Red dotted lines denote the deleted segments. Lengths of deletions/insertions is indicated on the left; (**E**) The permeability of the anterior PM of Bm01504-KO transgenic silkworm was evaluated using FITC-dextran (500 kDa) and fluorescence microscopy (100×). White lines show the localization of the PM. Ex, External; In, Internal.

We designed two small guide RNA (sgRNA) targets of *Bm01504* by using CRISPR direct (https://design.synthego.com/). Bm01504-sgRNA1 and Bm01504-sgRNA2 were designed and cloned to form the recombinant piggyBac (3×P3-DsRed-SV40+U6-Bm01504-sgRNA1+U6-Bm01504-sgRNA2) vector (Figure 5A). The recombinant vector and the helper vector were co-injected into 305 embryos, and the sgRNA transgenic silkworms with red eyes (Bm01504-sgRNA, Figure 5B) were generated, as described above for Bm01504-OE. Inverse PCR findings revealed only one insertion site located in the intergenic region on chromosome 2 (Figure 5C). Bm01504-sgRNA transgenic silkworms with red eyes were hybridized with Cas9 transgenic silkworms with green eyes, then Bm01504-knockout (Bm01504-KO) transgenic individuals with both red and green eyes were screened from their offspring (Figure 5B). sgRNA1 was found to be located at two positions because *Bm01504* contained many repeats. A large deletion of *Bm01504* found by PCR amplification and sequencing confirmed that *Bm01504* was knocked out (Figure 5D). The PM permeability of Bm01504-KO individuals was evaluated using FITC-dextran. The results showed that the 500 kDa dextran did not pass through the PM of WT, whereas it did pass through the PM of Bm01504-KO individuals (Figure 5E), indicating that Bm01504-KO transgenic silkworms increased the permeability of the PM.

### 2.5. Economic Characteristics of Transgenic Silkworm Lines

**Figure 6:**
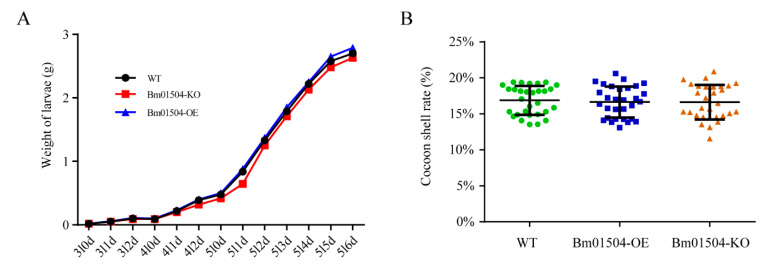
Economic characteristics of Bm01504-KO, Bm01504-OE, and WT. (**A**) Weight changes in the transgenic lines and WT from the third instar to 6th day of the fifth instar. The average weight of 30 larvae per group is shown. 3l, Third instar of larval stage; 4l, Fourth instar of larval stage; 5l, Fifth instar of larval stage; nd, Day n; (**B**) analysis of the cocoon shell rate of the transgenic lines and WT at pupal stage. Thirty cocoons of each silkworm strain were randomly selected on 3rd day in the pupal stage, and the cocoon shell rate was calculated. Each value represents the average of three repeated measurements. The experiments were repeated thrice.

To assess the effects of knocking out and overexpressing *Bm01504* on the growth status and economic characters of silkworms, changes in the body weight at different developmental stages and the cocoon rate at pupal stage were analyzed in the transgenic lines. The results showed that the average weight of the third instar larvae was approximately 0.02–0.1 g, the fourth instar larvae weighed 0.1–0.4 g, the fifth instar larvae weighed 0.4–2.8 g, and no significant differences in average weight were observed among Bm01504-KO, Bm01504-OE, and WT (Figure 6A). The cocoon shell rate ranged from 12% to 24% in the Bm01504-KO, Bm01504-OE, and WT, with no significant differences among them (Figure 6B).

### 2.6. Transgenic Larvae have Different Levels of Resistance to BmNPV

**Figure 7:**
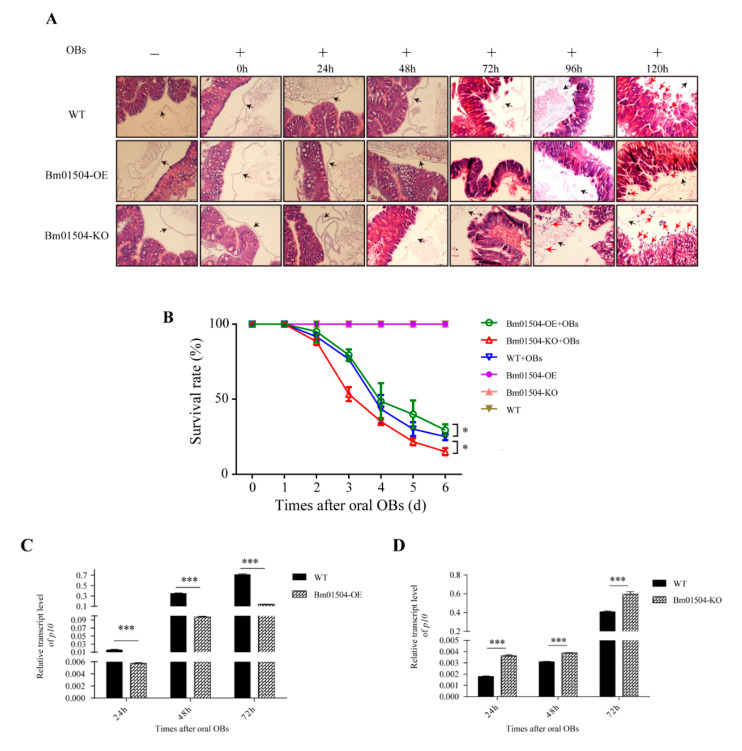
Anti-*B. mori nucleopolyhedrovirus* (BmNPV) activity of Bm01504-OE/ Bm01504-KO. (**A**) Paraffin sections of anterior midgut of transgenic silkworm infected with oral occlusion bodies (OBs) at different time. The midgut cells of Bm01504-OE, Bm01504-KO, and WT without oral OBs were orderly arranged and the PM structure was intact, and there was no difference among Bm01504-OE, Bm01504-KO, and WT. The structure of the midgut and PM did not change in Bm01504-OE, Bm01504-KO, and WT after oral OBs (0–24 h). However, the PM of Bm01504-KO was damaged in the following hours (48–120 h), with disordered midgut cells (48–72 h) and gradual nuclei loss (96–120 h). The PM of Bm01504-OE and WT were also damaged, but at later time points than Bm01504-KO (72–120 h), with similar disordered midgut cell arrangements being observed in both Bm01504-OE and WT (72–96 h). A smaller number of the nuclei were lost in WT compared with Bm01504-KO (120 h), and to an even lesser extent in Bm01504-OE than in WT. Black arrows show the location of the PM and red arrows show the exfoliated nuclei; (**B**) Survival rates of the transgenic silkworm larvae after infection with oral OBs. Each experiment included OB -infected or non-infected Bm01504-OE, Bm01504-KO, and WT silkworms (*n* = 30 per group); (**C**,**D**) Analysis of *p10* transcript levels in Bm01504-OE, Bm01504-KO and WT silkworms at 24, 48, and 72 h after infection with oral OBs d, Day; *p10*, late expressed gene in BmNPV. * *p* < 0.05, *** *p* < 0.001.

Paraffin sections showed that the midgut cells of Bm01504-OE, Bm01504-KO, and WT without BmNPV infection were orderly arranged and the PM structure was intact. The structure of the midgut and PM did not significantly differ among Bm01504-OE, Bm01504-KO, and WT (Figure 7A). Next, structural changes in the midgut and PM were evaluated on 1st day of the fifth instar in larvae infected with BmNPV via oral occlusion bodies (OBs). The structure of the midgut and PM did not significantly change among Bm01504-OE, Bm01504-KO, and WT at 0–24 h (Figure 7A). When the larvae were orally infected with OBs for 48 h, the arrangement of the midgut cells in Bm01504-KO became disordered and the structure of the PM was destroyed, whereas the midgut cells of Bm01504-OE and WT remained normal and neatly arranged (Figure 7A). When the larvae were orally infected with OBs for 72 h, the midgut cells were disordered in Bm01504-KO, with dilated nuclei, some of which became detached. At this point, the midgut cells of WT and Bm01504-OE also became disordered and the PM structure was destroyed. Bm01504-KO larvae infected with OBs for 96–120 h showed loss of many nuclei in the midgut, and the cells were more severely damaged (Figure 7A). WT and Bm01504-OE larvae were orally infected with OBs for 96 h showed dilated midgut cells with disordered nuclei, and, upon 120 h, the midgut cells of Bm01504-OE were loosely arranged with nuclear detachment, but to a lesser extent than in the WT group (Figure 7A). These results suggested that BmNPV is more likely to infect the midgut of Bm01504-KO, whereas Bm01504-OE somewhat delays damage to the midgut caused by BmNPV.

To confirm the anti-BmNPV capacity of Bm01504-OE and Bm01504-KO transgenic lines, the 1st day of the fifth-instar larvae of the transgenic lines were orally infected with OBs. Each larva was fed 1 × 10^8^ OBs. The survival rates of the WT, Bm01504-OE, and Bm01504-KO upon 6 days of infection were 25.0%, 29.4%, and 15.0%, respectively (Figure 7B). The survival rates of Bm01504-KO and Bm01504-OE were significantly lower and higher, respectively, than that of the WT group (Figure 7B). In addition, qRT-PCR data showed that the levels of *p10* at 24, 48, and 72 h after infection with oral OBs were significantly lower and higher in Bm01504-OE and Bm01504-KO, respectively, than in the WT (Figure 7C,D). These results suggested that Bm01504-OE enhanced, whereas Bm01504-KO decreased resistance to BmNPV.

## 3. Discussion

A total of 40 proteins encoding the peritrophin-A domain were identified in the silkworm genome database using bioinformatics analysis, including CHTs (*n* = 3), MUCs (*n* = 4), CDAs (*n* = 3), CPAP 1 (*n* = 13), CPAP3 (*n* = 6), and peritrophins (*n* = 11). Li et al. (2019) had previously identified 46 proteins encoding the peritrophin-A domain in the silkworm genome database, including 15 peritrophins [20]. These differences may result from potential false positives and ambiguous data, which further analysis herein performed have corrected. Phylogenetic analyses of CBD of peritrophins from silkworm and other species revealed that many of the CBDs from the same protein tended to cluster together, suggesting that CBD appeared before insect species differentiation. However, the events that led to the increased numbers of CBDs in peritrophins most probably occurred after insect species diversification. Interestingly, these results were in agreement with previously reported findings on *Manduca sexta* and *Tribolium castaneum* [17,18]. Unlike the evolutionary characteristics of other peritrophins, two CBDs from Bm09641 and McPMP2 of *Mamestra configurata* were clustered together in an evolutionary pattern similar to that of CPAP3. Three CBDs in CPAP3 from various insect species clustered together in three different groups. This indicated that CPAP3s evolved to become a multiple CBDs family prior to the divergence of species [17]. Furthermore, there was 45% and 99% similarity between Bm01504 and Bm01491, and between Bm14488 and Bm01361, respectively, indicating that peritrophins may be formed from old genes after species differentiation. Altogether, these results suggest that peritrophins have evolutionary diversity. Multiple sequence alignment showed that CBD comprised 50–56 amino acid residues, with six highly conserved cysteine residues. The amino acids between cysteine residues were CX_11-15_CX_5_CX_9-14_CX_11-12_CX_6-7_C. Similarly, Jasrapuria et al. (2010) described that the CBD in peritrophins of *Tribolium castaneum* comprised 52–56 amino acid residues, and that the features of conserved cysteines and amino acids comprised CX_11-17_CX_5_CX_9-14_CX_12_CX_6-7_C [18].

Our qRT-PCR findings revealed that *Bm09641* and *Bm01504* (with 2 and 4 CBDs, respectively) were expressed in the anterior midgut, whereas *Bm00185*, *Bm01491*, and *Bm11851* (with 8, 16, and 1 CBDs, respectively) were expressed throughout the midgut. Of note, the expression of peritrophins in the midgut was only detected on 3rd day of the fifth instar, but this expression pattern may change within different developmental stages. Peritrophins in *Tribolium castaneum* containing 1–3, 2–5, and 5–14 CBDs were mainly distributed in the anterior, middle, and posterior midgut, respectively [41], which further suggests that peritrophins with different amounts of CBDs are expressed in different parts of the midgut, and the distribution of peritrophins in the midgut is diverse among insect species. The developmental expression profiles showed that peritrophins were expressed more during the feeding period than in the molting stage, and the expression of peritrophins gradually increased along with developmental process. The expression of *Bm11851*, *Bm01504*, *Bm00185*, and *Bm01491* decreased at 7th day of the fifth instar. Rodríguez-de la Noval et al. (2019) detected *SfPER* transcripts in the midgut from the larval to adult stages of *Spodoptera frugiperda*, revealing that *SfPER* mRNA levels considerably increased from L3 to L6 larval instars before decreasing to almost undetectable levels in pre-pupae [8]. The spatial expression profiles of two peritrophin-like genes (*peritrophin-57* and *peritrophin-37*) in the common cutworm, *Spodoptera litura,* was similar to that of *SfPER* [23]. PM is produced in the midgut during actively feeding stages of the life cycle. As a PM structural protein, the regulation of peritrophins expression is tightly correlated with PM and midgut. Therefore, these results indicate that peritrophins play an important role in food intake and digestion. However, the expression of *Bm09641* increased on 7th day of the fifth instar. We inferred that *Bm09641* not only affects food digestion and absorption in larvae, but also plays an important role in metamorphosis. In summary, peritrophins play an important role in the growth and development of insects.

The PM is located between food and midgut epithelial cells, and the permeability of the PM is important for its function, serving as an ultrafilter regulating the exchange of nutrients and digestive enzymes, retaining undigested food particles, microorganisms and toxins in the endoperitrophic space [44,45]. The enhancement of NPV infection occurs, since entomopoxvirus (EPV) spindles lead to the disintegration of the PM as a barrier against NPV virions [46]. Genetic evidence for a protective role of the PM against intestinal bacterial infection has been observed in *Drosophila melanogaster* [47]. In addition, Agrawal et al. (2014) found that RNAi, for *TcPMP3* and *TcPMP5-B* in *Tribolium castaneum*, 2 MDa dextrans penetrated the epithelium of the median midgut, whereas dextrans were completely retained within the PM lumen of control larvae, indicating the loss of structural integrity and barrier function of the larval PM. Ultimately, the loss resulted in the depletion of the fat body, growth arrest, molting defects, and mortality in *Tribolium castaneum* [41]. Oliveira et al. (2019) also found that bees fed on anti-peritrophin-55 antibody showed an increase in peritrophic matrix permeability, but their survival was not affected [44]. The pupal weight and adult emergence rate of *Spodoptera frugiperda* decreased after interfering with *SfPER* (3 CBDs), possibly due to structural alterations in the PM that impaired digestion [8]. Dimopoulos et al. (2017) found a significant increase in *Enterobacteriaceae* load in the PM disrupted cohorts silenced by RNAi of the *APER1* gene that encodes peritrophin [48]. These findings showed that the peritrophins plays a vital role in maintaining the structure and function of PM.

Baculoviruses are insect-specific, enveloped viruses with circular, supercoiled double-stranded. BmNPV is a member of the family [49]. To investigate the antiviral effect of *Bm01504* against BmNPV infection in the silkworm, *Bombyx mori*, we generated Bm01504-OE and Bm01504-KO transgenic silkworms via embryo microinjection. Permeability assays using FITC-dextran showed that Bm01504-KO transgenic silkworms increased the permeability of the PM, whereas Bm01504-OE transgenic silkworms reduced PM permeability. These observations indicated that *Bm01504* can change the structure of the PM. Furthermore, we found that Bm01504-OE transgenic silkworms had enhanced resistance to BmNPV, whereas Bm01504-KO transgenic silkworms had decreased resistance to BmNPV. In summary, *Bm01504* plays an important role in the resistance of silkworms to BmNPV invasion. Baculoviruses produce two phenotypes: occlusion derived virus (ODV) and budded virus (BV). These two types of virions contain the same genome but differ in the morphogenesis and composition of their envelopes and their functions in the virus life cycle. The ODVs are enclosed in a paracrystalline protein (polyhedrin or granulin) matrix forming an occlusion body (OB). The alkaline environment of the midgut triggers the dissolution of polyhedra (OB) and the release of ODV. Once released, ODV can damage the PM to gain access to the midgut epithelium [50]. We speculated that the PM permeability of Bm01504-OE transgenic silkworms was decreased, thus preventing ODV from passing through the PM and entering the midgut. However, the PM permeability of Bm01504-KO transgenic silkworms was increased, which facilitated ODV passage through the PM to penetrate the midgut.

The white spot syndrome virus (WSSV) is a major pathogen that infects shrimp in cultures. Previous studies have found LvPT that are similar to insect peritrophins in *Litopenaeus vannamei* could interact with VP37 in WSSV [36]. We hypothesized that Bm01504 might interact with viral proteins to inhibit exogenous pathogenic microorganisms from infecting the midgut through PM. The mechanism needs further investigation. Although we found that *Bm01504* can resist invasion of BmNPV by maintaining the structural integrity of the PM, whether *Bm01504* has other mechanisms to resist pathogenic microorganisms invasion remains to be elucidated.

## 4. Materials and Methods

### 4.1. Silkworm Strain and Virus

Silkworm strain *305* was a gift from the Sericulture and Agri-food Research Institute, Guangdong Academy of Agricultural Sciences (Guangdong, China). The larvae were reared on fresh mulberry leaves at 25 ± 2 °C with 75% relative humidity under a photoperiod of 12 h light/12 h dark. Fourth instar silkworm larvae were fed mulberry leaves smeared with OBs, then hemolymph containing BmNPV was obtained from infected silkworms. The numbers of OBs were counted by hemocytometry and stored at 4 °C.

### 4.2. Identification of Peritrophins in the Bombyx mori Genome Database

We downloaded the Hidden Markov Model (HMM) containing the CBM_14 family from the PFAM database (http://pfam.janelia.org/). PfamID: PF01607. Based on this model, proteins containing CBD were searched in the silkworm genome database (http://www.silkdb.org/silkdb/). We searched candidate proteins in the Pfam database individually to reduce the number of false positive results.

### 4.3. Phylogenetic Analysis and Multiple Sequence Alignment

Sequences of multiple proteins were aligned using the MUltiple Sequence Comparison by Log-Expectation (MUSCLE) (https://www.ebi.ac.uk/Tools/msa/muscle/) and GENEDOC (https://genedoc.software.informer.com/27/) software. Multiple sequences were aligned using MUSCLE software before performing phylogenetic analysis. A phylogenetic tree was constructed by the neighbor-joining method using MEGA X software [51]. The branch strength of the phylogenetic tree was assessed using a bootstrap analysis of 2000 replications [17,20].

### 4.4. Oral Inoculation of OBs

Fresh mulberry leaves were cut into 1 × 1-cm squares, which were then smeared with 1 × 10^8^ OBs. 1st day of the fifth-instar larvae were given mulberry leaves smeared with OBs (one piece per larva). We started timing after the viral mulberry leaves were consumed, and then provided the silkworms with fresh mulberry leaves.

### 4.5. Semi-Quantitative RT-PCR and qRT-PCR

Total RNA was purified from each sample by using TRIzol reagent (Thermo Fisher Scientific Inc., Waltham, MA, USA) and 2 µg of mRNA was reverse transcribed into cDNA. Peritrophins expression at different developmental stages and in tissues was detected using semi-quantitative RT-PCR. The internal control was *Actin 3*. The mRNA levels of the genes were determined by qRT-PCR using SYBR™ Select Master Mix (Bio-Rad, Hercules, CA, USA). The internal control was eukaryotic translation initiation factor 4A (microarray probe ID: sw22934). The experiment was repeated three times with biological and technical replicates. Table 2 lists all the primers used.

### 4.6. Vector Construction

Bm01504-OE vector: The full-length coding sequence (CDS) of *Bm01504* was amplified using Bm01504 (cds) primers with BamH I and EcoR I restriction enzyme splice sites, and cloned into the pMD19-T vector (Takara, Dalian, China). The *Bm01504* gene was spliced from pMD19-Bm01504 by BamH I and EcoR I, and connected to the intermediate vector psl1180 (Opie2-MCS-SV40) to form psl1180 (Opie2-Bm01504-SV40). The expression cassette (Opie2-Bm01504-SV40) was spliced from psl1180 (Opie2-Bm01504-SV40) by Asc I and connected to piggyBac (3×P3-DsRed-SV40) to form piggyBac (3×P3-DsRed-SV40+Opie2-Bm01504-SV40).

Bm01504-sgRNA vector: Using U6-F1 and Bm01504-sgRNA1-R1 as primers and plasmid pMD19-T-U6 as a template for PCR amplification. The PCR product was diluted 50-fold and then used as the template for a secondary PCR with primers U6-F1 and U6-R2. The product of this reaction was diluted 50-fold and then used as the template for a tertiary PCR with primers U6-F1 and U6-R31, containing BamH I and EcoR I restriction enzyme sites, respectively. Consequently, the expression cassette (U6-Bm01504-sgRNA1) was obtained. Similarly, we obtained the U6-Bm01504-sgRNA2 expression cassette via multiple rounds of PCR by using the primer pair U6-F2 and Bm01504-RNA2-R1 followed by U6-R2 and U6-R32 (with Not I and Xba I splice sites, respectively). This cassette was cloned into pMD19-T. Plasmid pMD19 (U6-Bm01504-sgRNA1) and pMD19 (U6-Bm01504-sgRNA2) were digested using BamH I and EcoR I, and Not I and Xba I, respectively. The expression cassette was connected to the intermediate vector psl1180, then (U6-Bm01504-sgRNA1+U6-Bm01504-sgRNA2) spliced from psl1180 using BamH I and Xba I. The digested products were flattened and ligated to piggyBac (3×P3-DsRed-SV40) to form piggyBac (3×P3-DsRed-SV40+U6-Bm01504-sgRNA1+U6-Bm01504-sgRNA2). The sequence of the recombinant vector was verified at the Beijing Genomics Institute. All the primers used in the vector construction are shown in Table 3.

### 4.7. Microinjection and sCreening

The transgenic vectors, piggyBac (3×P3-DsRed-SV40+Opie2-Bm01504-SV40) and piggyBac (3×P3-DsRed-SV40 +U6-Bm01504-sgRNA1+U6-Bm01504-sgRNA2), were mixed with the A3 helper vector, then injected into silkworm eggs within 3 h of spawning using a microinjector (Eppendorf, Hamburg, Germany). Hatched larvae were bred as generation G0 and reared at 25 °C. G1 silkworms were produced by backcrossing of G0 silkworms. Transgenic G1 silkworms were screened using a fluorescence stereomicroscope (Leica, Wetzlar, Germany) and identified by their DsRed positive compound eyes.

### 4.8. Analysis of Insertion Site

The genomic DNA of Bm01504-OE and Bm01504-sgRNA transgenic silkworms was extracted and then digested with Hae III (New England Biolabs Inc., Ipswich, MA, USA) for 12 h at 37 °C. The DNA fragments were cyclized withSolution I (Takara Bio Inc., Kusatsu, Japan). The products were used for inverse PCR using the primers pBac L and pBac R, as described by Liu et al. (2016) [49]. Then, the fragments were cloned into the pMD19-T vector and sequenced. The results were analyzed using SilkBase (http://silkbase.ab.a.u-tokyo.ac.jp/cgi-bin/index.cgi).

### 4.9. FITC-Dextran Permeability Assays

Larvae at 3rd day of the fifth instar were fed with mulberry leaves that were smeared with FITC-dextran of different molecular masses (Sigma-Aldrich Corp., Saint Louis, MO, USA). The anterior midgut was dissected and fixed in 4% paraformaldehyde overnight at 4 °C. Tissues were embedded in OCT (Cell Path, UK) and sectioned using a frozen slicer (Thermo Scientific Inc., Waltham, MA, USA) at −24 °C. Transverse 20-µm sections were analyzed using fluorescence microscopy (Olympus, Tokyo, Japan).

### 4.10. Analysis of Economic Characteristics

A total of 30 larvae of each silkworm strain were screened at various developmental stages, from the third instar to 6th day of the fifth instar. The average weight of the larvae for each developmental stage was calculated. A total of 30 cocoons of each silkworm strain were randomly selected on 3rd day of the pupal stage, and the cocoon shell rate was calculated. Each transgenic line was assessed using three independent replicates.

### 4.11. Paraffin Sectioning

The anterior midgut was dissected and fixed in 4% paraformaldehyde overnight at 4 °C, dehydrated in a graded ethanol series, embedded in paraffin, and sectioned at 5 μm thickness by using a paraffin slicer (Leica, Wetzlar, Germany). The slices were stained with hematoxylin and eosin and examined by light microscopy (Olympus, Tokyo, Japan).

### 4.12. Statistical Analysis

Significant differences were analyzed using *t*-test. Values with *p* < 0.05 were considered statistically significant. Data from three independent experiments are presented as means ± SEM.

## 5. Conclusions

In this study, we identified 11 peritrophins in the silkworm, *Bombyx mori*. We found that five peritrophins (*Bm01504*, *Bm09641*, *Bm01491*, *Bm00185*, *Bm11851*) were expressed in the midgut. Further study found that *Bm01504* was able to participate in the resistance to BmNPV. Our study provides a new dimension of peritrophins function, and the results should be benefit for further functional studies on the silkworm resisting pathogenic microorganisms.

## Figures and Tables

**Table 1:** List of the peritrophins detected in the genome of *Bombyx mori.*

Gene Name	Gene ID ^1^	Gene ID ^2^	Chr.	Protein Length (Number of aa)	Number of CBDs
*Bm01504*	BGIBMGA001504	KWMTBOMO12505	21	392	4
*Bm00185*	BGIBMGA000185	KWMTBOMO00962	2	1261	8
*Bm03115*	BGIBMGA003115	KWMTBOMO02225	4	561	6
*Bm07250*	BGIBMGA007250	KWMTBOMO10355	17	1527	11
*Bm01491*	BGIBMGA001491	KWMTBOMO12463	21	1878	16
*Bm07902*	BGIBMGA007902	KWMTBOMO08750	15	190	1
*Bm09641*	BGIBMGA009641	KWMTBOMO00941	2	225	2
*Bm01361*	BGIBMGA001361	KWMTBOMO00967	2	340	2
*Bm14488*	BGIBMGA014488	KWMTBOMO00966	2	282	1
*Bm11851*	BGIBMGA011851	KWMTBOMO06502	11	539	1
*Bm01010*	BGIBMGA001010	——	13	241	1

^1^ Retrieve Old GeneModel (2008); ^2^ Retrieve RNA-seq and GeneModel (2017). Chr, Chromosome; aa, amino acids; CBD, Chitin binding domain.

**Table 2:** Primers used for polymerase chain reaction (PCR).

	Name	Forward (5′→3′)	Reverse (5′→3′)
Primers for semi-quantitative RT-PCR	*Bm01504*	TGGCCTCAGAATGTCGACT	CAATAATCTAAAATCCATAATGCTAC
	*Bm00185*	CATCCTCCCCTGGGCTCAC	CGTAATCAAGGTCATTTGTTCGC
	*Bm03115*	ACCTGTTATGAACCCGTTTGTGC	CGTTCACATTCTGGACCGCC
	*Bm07250*	TAACAGAGCAATCTACAAATCAAGC	TGGTGGTAGAAGATTCAGTGCC
	*Bm01491*	CGTCAATACTGGTCCTTGTAACTGT	GTAGTGTCTGATGTTTTGTCGTGC
	*Bm07902*	TCGGACCTCGCATAGCAGC	TTTCAGCGTAATCGCAGTAGCC
	*Bm09641*	ATGTTAGGTAAAGCCCTTAGTCTCTTG	CTAGTTTTTGTACACAATGAATTCGC
	*Bm01361*	CATCGGCGAGACAAGAGGT	AGTGCGAAGGCGGTATCC
	*Bm14488*	CATCGGCGAGACAAGAGGT	AGTGCGAAGGCGGTATCC
	*Bm11851*	CCTTGTGGCTCCTGTGTTG	CAGTATCAGTGCCTTCTTCGTC
	*Bm01010*	GGTCCGTATGGATTTATTTGCGA	ATGCCACTCCAGTTCTGTGTAAAAA
	*Actin3*	AACACCCCGTCCTGCTCACTG	GGGCGAGACGTGTGATTTCCT
Primers for qRT-PCR	*Bm09641*	CTGAAGGTTCGGGCTTGGGT	TGTGCCTGCTGAGTCTGCTGTG
	*Bm01504*	TGGCCTCAGAATGTCGACT	CAATAATCTAAAATCCATAATGCTAC
	*Bm00185*	CATCCTCCCCTGGGCTCAC	CGTAATCAAGGTCATTTGTTCGC
	*Bm11851*	GCAGAACAGGTTTGCGACTG	GCTCAGGCTCTTGTTCTGGT
	*Bm01491*	AAAGCTCCAGGGAGACAACG	TCCTCACCTGGAACGACTCT
	*sw22934*	TTCGTACTGGCTCTTCTCGT	CAAAGTTGATAGCAATTCCCT
	*p10*	TAGACGCCATTGCGGAAA	CGGGCAAACCGTCCAAA

**Table 3:** Primer sequences for vector construction.

Name	Primer (5′→3′)
Bm01504 (cds)-F	gatccATGGAAAAATTTAAAGGTATTCTATTAGTATTATAC
Bm01504 (cds)-R	gaattcCATGGAATAATTCTTGAACCGCAGT
U6-F1	ggatccAGGTTATGTAGTACACATT
Bm01504-sgRNA1-R1	GCTATTTCTAGCTCTAAAACTTTCCAGTGTTATCGTTTTCACTTGTAGAGCACGATATT
U6-R2	CAAGTTGATAACGGACTAGCCTTATTTTAACTTGCTATTTCTAGCTCTAAAACA
U6-R31	gaattcAAAAAAGCACCGACTCGGTGCCACTTTTTCAAGTTGATAACGGACTAG
U6-F2	gcggccgcAGGTTATGTAGTACACATT
Bm01504-RNA2-R1	GCTATTTCTAGCTCTAAAACCCAAATCTGTTGTATAATCCACTTGTAGAGCACGATATT
U6-R32	tctagaAAAAAAGCACCGACTCGGTGCCACTTTTTCAAGTTGATAACGGACTAG

## Supplementary Materials

Supplementary materials can be found at https://www.mdpi.com/1422-0067/21/21/7973/s1.

## Abbreviations

Bm01504-OE	Bm01504 overexpression
Bm01504-KO	Bm01504 knockout
WT	Wild type
CBD	Chitin binding domain
BmNPV	*B. mori nucleopolyhedrovirus*
PM	Peritrophic membrane
PMP	Peritrophic membrane protein
CBM_14	Carbohydrate Binding Module family14
ChtBD2	Type 2 CBD
CPAP	Cuticle proteins analogous to peritrophin
CHT	Chitinase
CDA	Chitin deacetylase
MUC	Mucin
MD	Mucin domain
HMM	Hidden Markov model
mRNA	Messenger RNA
RT-PCR	Reverse transcription polymerase chain reaction
qRT-PCR	Quantitative RT-PCR
FITC	Fluoresceine isothiocyanate
DsRed	*Discosoma* sp. red fluorescent protein
Opie2	*Orgyia pseudotsugata multicapsid nucleopolyhedrovirus* (OpMNPV) immediate-early 2 (ie2)
SV40	Terminator of Simian virus 40
PAM	Protospacer adjacent motif
ODV	Occlusion-derived viruses
BV	Budded virus
OB	Occlusion body
WSSV	White spot syndrome virus
MUSCLE	Multiple sequence comparison by log-expectation
CDS	Coding sequence
