# Supplementary material for: Identification of Peritrophins and Antiviral Effect of Bm01504 against BmNPV in the Silkworm, Bombyx mori
Source: Int J Mol Sci. 2020 Oct 27;21(21):7973. doi: 10.3390/ijms21217973 (PMC7663561; doi:10.3390/ijms21217973)
Supplement: Supplementary file 1 [file ijms-21-07973-s001.pdf]

Table S1. Genes that contain the perirophin-A domain based on Hidden Markov Model (HMM) of CBM_14 family

	Gene ID	Conserved domain		Gene ID	Conserved domain
Mucin	BGIBMGA001480-PA	Mucin domain+ CBM_14	CPAP1	BGIBMGA006381-PA	CBM_14
	BGIBMGA009809-PA	Mucin domain+ CBM_14		BGIBMGA006382-PA	CBM_14
	BGIBMGA009891-PA	Mucin domain+ CBM_14		BGIBMGA003270-PA	CBM_14
	BGIBMGA009892-PA	Mucin domain+ CBM_14		BGIBMGA003272-PA	CBM_14
Chitin deacetylase	BGIBMGA010573-PA	Polysacc_deac_1+ CBM_14		BGIBMGA003273-PA	CBM_14
	BGIBMGA006213-PA	Polysacc_deac_1+ CBM_14		BGIBMGA003773-PA	CBM_14
	BGIBMGA006214-PA	Polysacc_deac_1+ CBM_14		BGIBMGA010029-PA	CBM_14
Chitinase	BGIBMGA010240-PA	Glyco_hydro_18+ CBM_14		BGIBMGA010077-PA	CBM_14
	BGIBMGA006874-PA	Glyco_hydro_18+ CBM_14		BGIBMGA010281-PA	CBM_14
	BGIBMGA006989-PA	Glyco_hydro_18+ CBM_14		BGIBMGA001857-PA	CBM_14
Peritrophins	BGIBMGA000185-PA	CBM_14		BGIBMGA000298-PA	CBM_14
	BGIBMGA014488-PA	CBM_14		BGIBMGA000421-PA	CBM_14
	BGIBMGA007250-PA	CBM_14		BGIBMGA001052-PA	CBM_14
	BGIBMGA011851-PA	CBM_14	CPAP3	BGIBMGA007678-PA	CBM_14
	BGIBMGA007902-PA	CBM_14		BGIBMGA007899-PA	CBM_14
	BGIBMGA001491-PA	CBM_14		BGIBMGA007920-PA	CBM_14
	BGIBMGA001504-PA	CBM_14		BGIBMGA007677-PA	CBM_14
	BGIBMGA009641-PA	CBM_14		BGIBMGA007900-PA	CBM_14
	BGIBMGA003115-PA	CBM_14		BGIBMGA007901-PA	CBM_14
	BGIBMGA001010-PA	CBM_14			
	BGIBMGA001361-PA	CBM_14			

Table S2. Similarities of peritrophins in *Bombyx mori.*

	Bm001504	Bm001010	Bm007902	Bm003115	Bm001361	Bm000185	Bm007250	Bm001491	Bm011851	Bm014488	Bm009641
Bm001504	1.00	0.17	0.25	0.22	0.20	0.31	0.19	0.48	0.16	0.17	0.28
Bm001010	0.17	1.00	0.15	0.21	0.16	0.13	0.14	0.21	0.13	0.16	0.17
Bm007902	0.25	0.15	1.00	0.20	0.16	0.30	0.19	0.22	0.13	0.17	0.20
Bm003115	0.22	0.21	0.20	1.00	0.16	0.23	0.23	0.22	0.15	0.16	0.22
Bm001361	0.20	0.16	0.16	0.16	1.00	0.19	0.16	0.20	0.11	0.90	0.16
Bm000185	0.31	0.13	0.30	0.23	0.19	1.00	0.20	0.27	0.14	0.17	0.21
Bm007250	0.19	0.14	0.19	0.23	0.16	0.20	1.00	0.21	0.13	0.16	0.23
Bm001491	0.48	0.21	0.22	0.22	0.20	0.27	0.21	1.00	0.12	0.18	0.28
Bm011851	0.16	0.13	0.13	0.15	0.11	0.14	0.13	0.12	1.00	0.11	0.14
Bm014488	0.17	0.16	0.17	0.16	0.90	0.17	0.16	0.18	0.11	1.00	0.16
Bm009641	0.28	0.17	0.20	0.22	0.16	0.21	0.23	0.28	0.14	0.16	1.00

Table S3. Accession numbers of peritrophins used in phylogenetic analysis and multiple sequence alignment

Gene name	Accession no.	Species	Order	CBD (n)
PpPMP4	EU031912	*P. papatasi*	Diptera	4
AePMP5	AAL05409	*Aedes aegypti*	Diptera	5
LcPMP4	2211343A	*Lucilia cuprina*	Diptera	4
LcPMP3	P91745	*L. cuprina*	Diptera	3
AgPMP2	AF030431	*Anopheles gambiae*	Diptera	2
MdPMP2	DQ369743	*Mayetiola destructor*	Diptera	2
SlPMP6	KC108816	*Spodoptera litura*	Lepidoptera	6
SlPMP4	JQ730735	*S. litura*	Lepidoptera	4
McPMP2	GU596430	*Mamestra configurata*	Lepidoptera	2
LstiPMP8	FJ408730	*Loxostege sticticalis Linne*	Lepidoptera	8
TnPMP10	AY345125	*Trichoplusia ni*	Lepidoptera	10
TcPMP2-A	GU128099	*Tribolium castaneum*	Coleoptera	2
TcPMP2-B	GU128100	*T. castaneum*	Coleoptera	2
TcPMP2-C	GU128101	*T. castaneum*	Coleoptera	2
TcPMP3	GU128102	*T. castaneum*	Coleoptera	3
TcPMP5-A	GU128103	*T. castaneum*	Coleoptera	5
TcPMP5-B	GU128104	*T. castaneum*	Coleoptera	5
TcPMP9	GU128105	*T. castaneum*	Coleoptera	9
